# Effect of farmer socio-economic characteristics on extension services demand and its intensity of use in post-conflict Liberia

**DOI:** 10.1016/j.heliyon.2022.e12268

**Published:** 2022-12-15

**Authors:** Togba V. Sumo, Cecilia Ritho, Patrick Irungu

**Affiliations:** a Department of Agricultural Economics, University of Nairobi, Nairobi, Kenya; b Department of Agricultural Economics, University of Nairobi, Nairobi, Kenya

**Keywords:** Agricultural extension services, Demand, Heckpoisson, Liberia, Post-conflict, Smallholder farmer

## Abstract

Farmers' demand for and use of agricultural extension services in post-conflict countries is key not only to agricultural productivity but for economic transformation and maintenance of peace. This paper examined the effect of socio-economic characteristics of smallholder rice farmers on demand for extension services and the intensity of their use in Gibi District of Liberia. Multistage sampling technique was used in select 296 smallholder rice farmers. Descriptive statistics were used to compare farmers' socio-economic characteristics and the heckpoisson model was used to simultaneously estimate the effect of socio-economic characteristics on demand for extension services and the intensity of their use. The results showed that farm income, sale of crops and mobile phone ownership had significant effect on farmers' demand for extension services, while gender, cash-crop ownership, use of improved seeds, and awareness of extension services had significant effects on the intensity of their use. The study suggests that government implements programs that promote increased provision of needed farm inputs for greater use of extension services and encourages commercialization among farmers. Also, service providers should tailor their services to the farmers' socio-economic characteristics.

## 1 Introduction

Limited use of improved agricultural technologies, and inadequate extension services that facilitate their use are major threats to productivity improvement, food security and poverty reduction in Sub-Saharan Africa [SSA] (FAO, 2009; Ragasa et al., 2016). The low adoption of new technologies and low farm yields weaken efforts to increase productivity and is compounded by lack of credits, limited access to markets and unsustainable use of limited resources by smallholder farmers. Even though the demand for food in SSA is increasingly driven by population growth and urbanization (Mohanty, 2013), increasing productivity through agricultural transformation is needed to achieve food security, poverty reduction, and social stability (Pye-Smith, 2012; Tomich et al., 2019).

Experience from the Green Revolution in Asia shows that agricultural transformation could be achieved based on how new technologies are developed and used especially using a demand-driven approach (Janvry et al., 2002; Evenson and Gollin, 2003). Demand-driven extension underscores the need to make available services that meet the needs and priorities of farmers, even if the “invisible hand of the market” does not ensure that the quantity and quality of extension services demanded by farmers are adequately supplied (Birner and Anderson, 2007). According to Birner and Anderson (2007), the approach might better address the goal of making extension services meet the needs and priorities of farmers, even if they are unable to demonstrate demand that leads to an adequate supply of said needs and priorities as the term is defined in economics.

Investment in research and development (R&D) for the generation of new agricultural technologies and their dissemination to farmers play a crucial role in raising agricultural productivity (Mellor, 2017; Tiruneh et al., 2015). Agricultural extension services strengthen farmers' capacity to make decisions leading to optimal use of their resources (Otchia, 2014; Swanson and Rajalahti, 2010). The process involves influencing farmers attitude towards making appropriate farm-level decisions to adopt new technologies that improve productivity (Al-Zahrani et al., 2016). The adoption and use of technologies that address problems at the farm level are knowledge-driven (Zhou and Chandra Babu, 2015). Therefore, transfer of knowledge and skills, and ensuring the adoption of new technologies play a critical role in raising production, improving livelihood and maintaining peace (Piesse and Thirtle 2010).

In Liberia, agriculture is the primary source of livelihood for about 80 percent of the population, and the sector plays a vital role in improving the economy in the post-conflict period (Government of Liberia, 2017). It contributed about 36 percent to Gross Domestic Product (GDP) in 2017, and the food sub-sector contributed about 50 percent of agricultural GDP (Central Bank of Liberia, 2016; Tyson 2017). Rice, the leading staple food, is a vital component of this contribution and accounts for about 50 percent of adult calorie intake (Adeola, 2018). Per capita annual rice consumption is estimated at 108 kg, one of the highest in SSA when compared to 35 kg (kg) in Nigeria, 41 kg in Tanzania, 43 kg in Ghana, and 65 kg globally (FAO, 2020). The crop is mainly grown in the uplands of Liberia by an estimated 71% of the population using traditional technologies particularly the “slash and burn” method, and the quantity produced is not enough for household consumption (GOL, 2012).

Following the end of the 14-year civil conflict in 2003, the Government of Liberia and its partners implemented several strategies to transform the agricultural sector to spur economic growth. Among these strategies, the Agricultural Sector Rehabilitation Program (ASRP) was created in 2010 to rehabilitate existing research institutions, agro-processing facilities, and roads destroyed during the civil conflict (Peterson, 2016). The National Rice Development Strategy (NRDS) was initiated in 2012 to enhance farmers' capacity to increase rice yields through tailor-made solutions generated by R&D (Government of Liberia, 2012). In particular, priority was given to agricultural extension to facilitate the transfer of technologies and knowledge to improve productivity and promote peace and stability in rural areas. Consequently, the government formulated the National Agricultural Extension and Advisory Services (AEAS) policy to drive the transformation effort (Mcnamara et al., 2011; Moore and Harder, 2015). Despite these well-intentioned interventions and the high rice production potential of Liberia, rice yields are low at an estimated 1.2–1.6 metric tons per hectare (MT/ha) compared to neighboring countries: Cote d’Ivoire (2.6 MT/ha), Ghana (2.8 MT/ha), and Senegal (4.1 MT/ha) (FAO, 2019). As a result, the country imports more than one-third of its annual rice demand (FAO, 2019), draining the scarce foreign exchange reserves. Moreover, only less than 10% of rice farmers have access to extension services or are linked to new technologies; less than 5% use fertilizers and improved seeds (Lah et al., 2018; Ahn et al., 2020).

Although previous studies carried out in Liberia focused on extension delivery methods and the human resource capacity of extension staff (Lah et al., 2018; Moore, 2017; Moore and Harder, 2015), there is no empirical evidence of the effects of socio-economic characteristics on farmers on their demand for extension services and the intensity of their use in Gibi District, the largest rice-producing area in Margibi County of Liberia. Past studies show that an understanding of the effect of farmers' socio-economic characteristics on their demand for extension services and the intensity of their use contribute to improving the quality of extension service delivery and technology transfer for productivity growth among rural poor farmers. Furthermore, it leads to favourable attitude towards acceptance of new technologies and optimal utilization of farm inputs (Abdallah and Awal, 2016; Ragasa et al., 2013). It also exposes them to the effects of extension on farm yields and household welfare, as an incentive to demand more of the services to adopt new technologies (Tadesse, 2017; Wossen et al., 2017). However, the results of these past studies are country specific and given the heterogeneity of the countries and the parameter estimates may not be unique for addressing the numerous constraints to demand and use of extension services in Liberia, a post-conflict country with competing developmental priorities.

A farmer’s decision about whether or not to use extension services depends on his/her demand for said services. When demand for and use of extension services are sequentially related, an endogenous sample selection model is used to simultaneously estimate the parameters to remove bias compare to using two separate equations (probit and poisson models) which would lead to bias in the parameter estimates and generates misleading conclusions. Therefore, the objective of this study was to estimate the effect of smallholder farmers' socio-economic characteristics on their demand for extension services and the intensity of their use in Gibi District, Liberia. The rest of the article is organized as follow: section two explains the methodology used in addressing the key research objective, section three presents the key research findings and discussions and the final section presents the conclusion and policy recommendations.

## 2 Materials and methods

### 2.1 Theoretical framework

Farm households' decision to seek extension services is a behavioral response to the need to increase agricultural productivity based on its production objectives. If a household’s production and consumption decisions are assumed to be inseparable, then the agricultural household model (AHM) postulated by Singh et al. (1986) can be used to explain the underlying motivation to seek extension services. According to Taylor and Adelman (2003), the AHM considers that producing households consume farm output and market the surplus thus underscoring the inseparability and joint nature of production and consumption. The first-order condition for profit-maximizing households gives the demand for extension services as a function of input and output prices (Makau et al., 2016). Since household production and consumption decisions are inseparable, the desired level of extension services required for use by farm households are assumed to be affected by their socio-economic characteristics (Liverpool-Tasie and Lenis Saweda, 2014; Ricker-Gilbert et al., 2011). Previous studies have used AHM to estimate demand for agricultural inputs and support services including extension (Liverpool-Tasie and Lenis Saweda, 2014; Makau et al., 2016; Ricker-Gilbert et al., 2011). Following Singh et al. (1986) demand model for extension services can be expressed as:

(1) $$F_{E}=f\left(C,P_{E},P_{O},A,Z\right)$$

where $F_{E}$ represents the number of extension visits a farmer paid for to the service providers. $F_{E}$ is affected by the number of time the farmer sought extension services, denoted by C, price of extension services $P_{E}$ and price of farm output sold $P_{O}$. Variables A and Z are household’s socio-economic characteristics and fixed assets respectively.

### 2.2 Econometric estimation

Household demand for extension services and the intensity of their use were considered a two-stage decision-making process. In the first stage, the farmer chooses whether or not to seek extension services and in the second stage, the farmer decides how much of the service to use (intensity of use) contingent on the choice decision in the first stage. For those farmers who choose not to seek extension services optimal decision is observed as zero, rather than treating it as unobserved. However, it is impossible to use Heckman (1979) two-step sample selection model which treats the number of contacts as continuous rather than a count variable. In the current study, the dependent variable for the first stage is weather the respondent seek extension services or not and for the second stage is the number of extension contacts (a count variable). Heckpoisson model was implemented to assess the effect of socio-economic characteristics of smallholder rice farmers on their demand for extension services and the intensity of their use in the Gibi District. The model is a combination of Probit and Poisson regression models and was preferred due to its ability to simultaneously estimate parameters of binary and count data and correct for sample selection biases (Cameron and Kolstoe, 2020). The Heckpoisson model is estimated in two stages, a selection and a count. Following Waruingi et al. (2021), the model for the selection part is specified as follow:

(2) $$S_{i}=\left\{ \begin{array}{c} 1,if\;X_{i}^{,}\beta +\varepsilon_{1i}>0\\ 0,if\;otherwise \end{array}\right.$$

where $S_{i}$ is the binary indicator (0;1) showing whether the $i^{th}$ household demanded extension services or not; $X_{i}$ is a vector of predictor variables for the $i^{th}$ household, β the parameters to be estimated, and $\varepsilon_{i1}$ is the error term for the selection outcome assumed to have a bivariate normal distribution with zero mean and covariance matrix. The number of times a household uses extension services (${Intensity=Y}_{i}$) is only observed when the household demands extension services. Waruingi et al. (2021) also specified the count outcome (intensity of use) equation as follow:

(3) $$Y_{i}=X_{i}^{,}\beta +\varepsilon_{2i}$$

where $Y_{i}$ is the frequency of extension contacts illustrating intensity of use, $\varepsilon_{2i}$ is the error term for the count outcome assumed to have a bivariate normal distribution with zero mean and a covariance matrix and the other variables remain the same as in Eq. (2).

#### 2.2.1 Empirical models

The first stage of model, Eq. (2) used to estimate the determinants of demand for extension services (selection outcome, S) is specified as follow (Greene 2012).

(4) $$S=\beta_{0}+\beta_{1}Gen+\beta_{2}\;\mathrm{Exp}+\beta_{3}Dist+\beta_{4}CCrp+\beta_{5}MPh+\beta_{6}FSize+\beta_{7}Com+\beta_{8}Inc+\beta_{9}\;CropD+\varepsilon_{i}$$

The second stage of the model, Eq. (3), applied to assess the drivers of intensity of use of extension services where the intensity indicator (Y) is only observed if demand for the services manifests (S = 1) (Greene 2012) is fitted into the data as follow:

(5) $$Y=\beta_{0}+\beta_{1}Age+\beta_{2}Gen+\beta_{3}\;\mathrm{Exp}+\beta_{4}Dist+\beta_{5}Ccrop+\beta_{6}Mbph+\beta_{7}Fsize+\beta_{8}Imseed+\beta_{9}Aware+\varepsilon_{i}$$

The dependent variables analyzed in this study are demand for extension services (0;1) and number of extension contacts. Definition of the variables used in the Heckpoisson model along with their hypothesized signs are presented in Table 1.

**Table 1:** Explanatory variables used in the model and their hypothesized signs.

Variables	Variable definition	Expected sign
Age	Age of the household head in years	±
Income	Household monthly farm income in US dollars	+
Experience	Years of farming experience of household head	+
Years of formal Schooling	Number of years household head spent in formal school	+
Farm Size	Size of cultivated land in acres	+
Distance	Distance to the extension source in kilometers	-
Awareness	Aware of NGO-provided extension; 0 = Aware, 0 = Otherwise	+
Improved rice seed	Household use improved rice seed; 1 = improved seed; 0 = traditional	±
Cash Crops	Household grows cash crops; 1 = cash crop, 0 = otherwise	+
Gender	Gender of household head; 1 = Male, 0 = Female	±
Sale of rice	1 = if farmers the sold proportion rice yield; 0 = otherwise	+
Mobile Phone	1 = Household head own mobile phone, 0 = otherwise	
Household size	Total number of person in household	+

**Age of the household head** is hypothesized to have either positive or negative effect on farmers' decision to seek and use agricultural extension services. Gido et al. (2015) found a positive relationship between age and demand for extension services, while Abdallah and Awal (2016) found a negative relationship in Ghana.

The variable, **farm income** is measured as the total monthly income generated from farming and is a proxy for wealth. Farmers with higher incomes are more likely to seek and demand agricultural extension services as found by Nambiro et al. (2006). The study hypothesizes that higher farm income will have a positive effect on demand for extension services.

**Gender of the household head** plays an important role in the demand for extension services in developing countries like Liberia, where males are key decision-makers in most households. Ogato et al. (2009) noted that in male-headed households, males are more likely to seek and use new knowledge than females, while females only make decisions when the males are absent. Therefore, male farmers are hypothesized to have a higher demand for extension services than female farmers.

**Distance** to the nearest extension source is hypothesized to have a negative effect on demand for extension services because roads in rural parts of Liberia are in deplorable conditions. Mutambara et al. (2013) found that distance to veterinary services negatively induced farmers' demand for those services in Zimbabwe.

**Awareness** about the availability of new agricultural support services and technology induces farmers' decision to seek the services or adopt new technologies available. For example, Anang et al. (2015) found that awareness of credit availability increased farmers' access to agricultural microcredit in Ghana. It is therefore hypothesized that awareness of the existence of extension service will have a positive effect.

Cash crops are a major source of income and households growing them are more likely to demand agricultural extension services targeting higher income. Maonga et al. (2017) found cash crop ownership induced increased demand for extension services. The variable is therefore hypothesized to positive effect on farmers demand for and use of extension services.

**Farm Size** influences farmers' demand and use of extension services. If farmers have bigger farm size, they will have higher demand for the services. It hypothesized that large farm size induces farm households' demand for extension services positively. Studies by Abdallah and Awal (2016) and Wossen et al. (2017) found that farm size increased the demand for extension services and the intensity of their use.

Large **household size** means availability of labor for agricultural work and more need for food. For a household to maximize farm output to meet their needs, they are likely to demand and use more extension services. In this study, the variable is expected to have a positive effect on household decision to demand and use agricultural extension services. Tadesse (2017) found that household size positive effect on women’s access to poetry extension services in Ethiopia.

Gido et al. (2015) and Kiprotich et al. (2019) found a positive relationship between **years of formal education** and demand and use of agricultural supports services. Working with farmers with more years of formal education can be rewarding for extension services providers especially when introducing new concepts because the most educated farmers have the ability to understand, interpret and apply new information well than the less educated ones (Ragasa et al., 2013). Therefore, it is hypothesized that the variable will positively influence demand for and intensity of use of extension services.

**Crop diversification** is a viable strategy to strengthen farmers' resilience in agricultural production. Ouma et al. (2014) a positive effect of crop diversification on demand for extension services and new technologies such as improved seeds among farmers in Kenya. Crop Diversification knowledge provided by extension professionals helps farmers reduce risk of losing on their investments by adopting viable alternatives for crop production. Therefore, the variable is expected to have a positive effect on demand for extension services.

Nambiro et al. (2006), Wossen et al. (2017) and Makau et al. (2016) found that **mobile phones** ownership had a positive effect on demand for extension services and the quantity of fertilizers purchased, respectively. Mobile phone ownership will positively affect farmers' demand and intensity of use of extension services because farmers use the device to search for information that can help them improve their farming practices and arrange appointments with extension agents.

**The variable, use of improved rice seed**, a proxy for technology adoption, requires technical knowledge from extension agents to get optimum output. Studies have found a positive relationship between the use of improved seeds and farmers' demand for and use of agricultural extension services (Makau et al., 2016; Maonga et al., 2017; Ragasa et al., 2013). In this study, the variable was hypothesized to have a positive effect on farmers' demand for and use of extension services. Use of improved rice varieties.

**Experience** in farming enables farmers to evaluate the usefulness of extension services, thus increasing their demand for extension services and their number of contacts with extension agents. Gido et al. (2015) found that years of farming experience increased farmers' demand and use of agricultural extension services in Kenya and Nigeria, respectively. Therefore, it is expected that farming experience will a positively effect on farmers demand for and their use of extension services.

**Sale of rice:** The variable is an indication that farmers market a proportion of their crops for income generation. Farmers who generate income from crop sales are likely to seek extension services to improve their production. Studies have shown that sale of crops has an effect on farmers' demand for extension services in Zimbabwe and Kenya (Foti et al., 2007; Makau et al., 2016). Therefore, the variable is expected to increase farmer’s demand for extension services.

### 2.3 Study area

The study was carried out in Gibi District. The district is approximately 17,000 square kilometers and is located in Margibi County in Central Liberia. Gibi District is the major rice production area in Margibi County and one of six districts dominated by NGO extension programs in the country (Moore 2017; Murphy et al. 2016). The average annual rainfall ranges between 4400 and 4500 mm while the mean annual temperature is 26 °C and longer sunshine with humidity ranging between 85 and 95%, which makes it ideal for agriculture activities (GOL, 2008). Most of the land in the district is fertile, swampy, and about 30 m above sea levels. Most of the inhabitants of the district are engaged in small-scale mixed food and cash crop production for livelihood (GOL, 2008; 2017). Rice is the main food crop, followed by cassava grown on farms with an average size of 1.2 ha, while rubber, cocoa, coffee, and oil palm are the most common cash crops produced in the district (GOL, 2008).

### 2.4 Sampling and data collection

The study used a multistage sampling technique. In the first stage, Margibi County was purposively selected because it is one of the counties where smallholder farmers are predominantly engaged in rice production. In the second stage, Gibi District was selected because it is dominated by donor-funded NGO extension programs for smallholder farmers, including those producing rice. In the third stage, three townships, Peter’s Town, Wohn, and Yanquilee, with a high population of smallholder rice farmers in Gibi District were purposively selected. Finally, smallholder rice farmers were randomly selected from a list of all rice farmers in Gibi District constructed with the assistance of extension officers. A total of 296 farmers were interviewed comprising 144 farmers who accessed extension services and 152 farmers who did not access extension services at the time of the survey. Of this number, 86 were from Yanquilee (41 accessors and 45 non-accessors), 92 from Peter Town (48 accessors and 44 non-accessors) and 118 from Wohn (55 accessors and 63 non-accessors). Face-to-face interviews were conducted by trained enumerators using a pre-tested and semi-structured questionnaire to collect data on socio-economic and institutional characteristics of the farmers. Open Data Kit (ODK) was used for data collection, while STATA version 15 was used for data analysis.

## 3 Results and discussions

### 3.1 Comparison of socio-economic characteristics of smallholder rice farmers in Gibi District

A comparison of the socio-economic characteristics of households by access to extension services (Table 2) shows that the average age of farm household heads and their years of experience in rice farming were 44 and 15 years, respectively. On average, farmers operated a farm size of 1.4 ha and were located four kilometers away from an extension source. These characteristics were not significantly different between those who accessed extension services and those who did not. The mean age, farm size and distance to an extension source are similar to those reported by Liberia Institute for Statistics and Geo-Information Services [LISGIS] (GOL, 2017) and years of experience reported concurs with Roberts et al. (2017). However, those with access to extension had significantly higher monthly farm income suggesting that extension services imparted the livelihood of the farmers. The high farm income for extension accessors is likely due to improvements in crop production as a result of the services they received. The findings agree with Danso-Abbeam et al. (2014), who found that access to extension services increased maize farmers' income in Ghana.

**Table 2:** Selected socio-economic characteristics of smallholder rice farmers with and without access to extension services in Gibi District.

Variable	Farmers with Access (n = 144)	Farmers without Access (n = 152)	All farmers (n = 296)	
	Means			*t-ratio*
Age of household head (Years)	43.4	44.8	44.1	1.10
Farming experience (year)	14.5	15.4	15.0	0.76
Monthly farm (US$)	53.0	33.3	43.0	-4.14∗∗∗
Farm Size (ha)	1.4	1.3	1.4	-0.63
Distance to extension source (km)	3.9	4.1	4.0	0.44
Years of formal schooling	4.1	4.6	4.3	0.91
Household Size	6.4	6.8	6.6	1.34
	**Percentages**			***z-ratio***
Crop Diversification (Yes)	94.4	94.1	94.3	-0.14
Awareness of NGOs (Yes)	80.0	57.9	68.6	-4.07∗∗∗
Mobile phone (Yes)	46.5	59.2	53.0	2.19∗∗
Access to Improved seeds (Yes)	36.8	34.9	35.8	0.73
Producing Cash Crops (Yes)	51.4	53.9	52.7	0.44
Gender (Male)	81.9	82.9	82.4	0.21
Sale of rice (Yes)	21.5	9.8	15.0	-2.76∗∗∗

Note: ∗∗∗, ∗∗, ∗ Significant at 1%, 5% and 10% probability levels respectively.

About 94% of the rice farmers in Gibi District practiced crop diversification using locally consumed crops and approximately 69% were aware of the existence of NGO extension programs. Awareness of NGO extension services by rice farmers was significantly different based on access to extension services. This could probably be the reason for a greater access to extension services among proportion of the farmers who accessed extension services in the study area.

Further analysis of the results shows that more than half of the rice farmers owned a mobile phone. The difference in mobile phone ownership was statistically significant at 5% level across those who did not access extension services. On average, more than one-third of the rice farmers in the study area used improved rice seeds, and more than half produced cash crops.

There was no significant difference between the two categories. The finding is higher than the four percent (improved rice seed) and 33% (cash crops) reported by LISGIS (GOL, 2017) but lower than the 46.5% reported by Saysay et al. (2016). This is because most farmers still rely on traditional varieties or are not exposed to improved varieties and depend on cash crop production as a source of income.

About 82.4% of the rice farmers were male. The results indicate a gender gap in smallholder rice farming in Gibi District. This is so because most female-headed households do not have productive capital such as land to farm. The result is similar to Saysay et al. (2016), who reported 87% male involvement in farming in central Liberia. The degree of rice sale among rice farmers in Gibi District was low. Only 15 % of the farmers reported sale of portion of their farm yield. The finding shows that sale of rice increased by only three percentage point from the 12% reported by LISGIS (GOL, 2017). Categorically, about 22% of the accessing farmers sold rice compared to 10% of the non-accessing farmers. The difference was statistically significant at 1% level. The low degree of commercialization among rice farmers is likely because most of the farmers are subsistence-oriented.

### 3.2 Effect of socio-economic characteristics of rice farmers on their demand for extension services and the intensity of their use

The test for independence was performed to justify the use of Heckpoisson model, and the result shows that the Wald Chi-square statistic was significant at the 1% level, implying a strong explanatory power of the model. The Wald test of independent equations was significant at 1% level, justifying a rejection of the null hypothesis of zero correlation between the decisions to demand extension services and the intensity of their use. Table 3 presents the results of the Heckpoisson regression model. Columns 2–4 present the variables that had a significant effect on demand (selection equation), while columns 5–7 report variables that affect the intensity of use (outcome equation). The results of the selection equation show that mobile phone ownership, sale of rice, and monthly farm income had significant effect on farmers' demand for extension services. Additionally, the results of the outcome equation show that the intensity of use was significantly influenced by the gender of the household head, use of improved seeds, awareness of extension services, and cash crop ownership.

**Table 3:** Effects of socio-economic characteristics on demand for extension services and intensity of use.

Variables	Selection equation: Demand for extension services			Outcome equation: Intensity of use		
	Coef.	Robust Std. Err.	Marginal Effect	Coef.	Robust Std. Err.	Marginal Effect
Age				-0.003	0.004	-0.008
Gender (Male)	-0.074	0.203	-0.036	-0.212	0.090	-0.523∗∗∗
Years of formal schooling				-0.007	0.009	-0.016
Farming experience (yrs)	-0.004	0.008	-0.002	-0.004	0.006	-0.009
Distance to market (km)	-0.045	0.030	-0.015	0.004	0.014	0.009
Producing cash crop (Yes)	-0.060	0.155	-0.024	0.138	0.072	0.338∗
Farm size (ha)	0.043	0.043	0.018	-0.006	0.019	-0.014
Monthly farm income (US$)	0.008	0.002	0.027∗∗∗			
Crop diversification (Yes)	-0.235	0.316	-0.093			
Used improved seed (Yes)				0.268	0.083	0.658∗∗∗
Aware of NGO extension (Yes)				-0.242	0.090	-0.594∗∗∗
Sale of rice (Yes)	0.543	0.220	0.211∗∗			
Mobile phone (Yes)	-0.344	0.151	-0.137∗∗	0.029	0.071	0.071
Household size	0.047	0.032	0.093			
Constants	0.437	0.450		1.21	0.213	
Wald chi 2 (9) = 45.71						
Prob > chi 2 = 0.000∗∗∗						
Log pseudo likelihood =	411.51					

Note: ∗∗∗, ∗∗, ∗ Significant at 1%, 5% & 10% probability levels respectively.

Consistent with a priori expectation, household monthly farm income had a positive and significant effect on demand for extension services at 1% level. The result indicates that a U$1.00 increase in farm income increased the probability of farmers demanding extension services by 0.3%. This could be explained by the fact that farm households that generate more income will tend to increase production and therefore increase their interest to demand extension services. The finding concurs with Nambiroet al. (2006), who found that income generated from crop sales had a positive effect on access to extension services in Eastern Kenya.

The effect of mobile phone ownership on smallholder rice farmers' demand for extension services was negative and significant at 5% statistical level. The marginal effect indicates that if a household owns a mobile phone, the probability of seeking extension services reduced by 14.1% compared to those who do not own a mobile phone. The result was not expected. However, this relates to the fact that mobile phone coverage in the study area is low and was not used as the preferred channel to contact extension services providers. The finding is inconsistent with Wossen et al. (2017) who found that mobile phone ownership had a positive effect on access to extension services in rural Nigeria.

The sale of rice had a positive and significant effect on demand for extension services by smallholder rice farmers at the 10% level. This implies that if a farm household sold rice, the probability to demand extension services increases by 20.4%. A likely explanation is that households that depend on farm yield to generate income may be compelled to seek extension services to increase their farm yields. The result corroborates with the findings of Foti et al. (2007) that the commercialization of farm enterprises increased farmers' ability to seek fee-for-service extension services in Zimbabwe.

Gender of the household head had a significant negative effect on the intensity of use of extension services by smallholder rice farmers at 1% level. If a farm household was headed by a female the probability of utilizing extension services increased by 58.8% compared to their male counterparts. This is because females provide more substantial labor on smallholder farms thus increasing their chances to use more extension services compared to their male counterparts (Rapsomanikis, 2015). The result is contrary to Korir’s (2016) findings that being a male-headed household increased the chances of adopting integrated pest management components in Embu East Sub-County, Kenya.

The use of improved seeds had a positive and significant effect on the intensity of use of extension services at 1% level. The marginal effect indicates that access to improved seeds increased the probability of using extension services by 5.4%. This finding is plausible because farmers ability to utilized extension services is dependent on the use of required inputs such as improved seeds to maximize the benefit of the services. The result is in line with Ragasa et al. (2013) that the use of improved seeds had a positive effect on the intensity of use of extension services in Ethiopia.

Cash crops ownership had a positive and significant effect on the intensity of use of extension services by farmers at 10% level. If a farm household owns cash crops, the probability of using extension services increased by 32.8%. The increased use of extension services by farm households that owned cash crops could be linked to the fact that cash crop is an important source of income in the study area. Most farm households that own cash crops rely on income generate from it to pay for extension services. Additionally, cash crop is input-intensive and would require technical assistance from extension service providers for optimal yields. Maonga et al. (2017) made a similar observation that farmers who owned cash crops seek more agricultural support services in Malawi.

The variable, awareness of extension services, had a negative and significant effect on the intensity of use of extension services at 1% level, contrary to expectations. This means that a farm household being aware of extension services decreases the probability to use it by 0.6%. A plausible explanation is that farmers might be aware of the services in their locale but do not seek to access them because they are not knowledgeable about the benefits of using them or the services are rarely available. The result is inconsistent with the findings from Kiprotich et al. (2019) that awareness increased the utilization of baobab tree products in Kenya.

## 4 Conclusion and recommendations

Knowledge of the effect of socio-economic characteristics of smallholder rice farmers on demand for extension services and the intensity of their use is crucial for effective agricultural extension service delivery, and their utilization among farmers to enhance growth in productivity, improve livelihood, and reduce poverty. The study sought to estimate the effect of social economic characteristics of smallholder rice farmers on their demand for extension services and the intensity of use in post-conflict Liberia, particularly in the Gibi District. The Heckpoisson endogenous sample selection model was used to analyze the data.

The results revealed significant differences in farmers' socio-economic characteristics by access to extension services. For Instance, there exists low female participation in rice farming, low rice sale, and low use of improved seeds among rice farmers in Gibi District. The Heckpoisson model selection equation results revealed that mobile phone ownership, rice sale, and monthly farm income had significant and positive effects on farmers' demand for extension services. Further, the count data equation results revealed that gender, cash crop ownership, use of improved seeds, and awareness of extension services had significant effects on the intensity of their use of extension services.

Given that the use of improved seeds has a significant effect on the intensity of use of extension services, there is a need for policymakers and extension stakeholders to implement programs that will encourage agribusiness entrepreneurs to provide needed agriculture inputs that are affordable and sustainable. By doing so, smallholder farmers will use more of the available extension services. Lastly, the finding that farm income has a significant effect on farmers' demand for extension services is critical for the ongoing promotion of demand-driven extension services in Liberia. Therefore, policymakers should develop and implement programs that promote increased commercialization among farmers.

## Declarations

### Author contribution statement

Togba V. Sumo: Conceived and designed the experiments; Performed the experiments; Analyzed and interpreted the data; Contributed reagents, materials, analysis tools or data; Wrote the paper.

Cecilia Ritho; Patrick Irungu: Conceived and designed the experiments; Analyzed and interpreted the data; Contributed reagents, materials, analysis tools or data.

### Funding statement

This work was supported by the African Economic Research Consortium (AERC).

### Data availability statement

Data will be made available on request.

### Declaration of interest’s statement

The authors declare no conflict of interest.

### Additional information

No additional information is available for this paper.
